# The Small Molecule Wnt Signaling Modulator ICG-001 Improves Contractile Function in Chronically Infarcted Rat Myocardium

**DOI:** 10.1371/journal.pone.0075010

**Published:** 2013-09-12

**Authors:** Tomoyo Sasaki, Hyosook Hwang, Cu Nguyen, Robert A. Kloner, Michael Kahn

**Affiliations:** 1 Department of Biochemistry and Molecular Biology, Keck School of Medicine of University of Southern California, Los Angeles, California, United States of America; 2 The Heart Institute, Good Samaritan Hospital, Los Angeles, California, United States of America; 3 Division of Cardiovascular Medicine, Keck School of Medicine of University of Southern California, Los Angeles, California, United States of America; 4 Norris Comprehensive Cancer Research Center, University of Southern California, Los Angeles, California, United States of America; 5 Department of Nuclear Medicine, Chonbuk National University Medical School and Hospital, Jeonju, Jeonbuk, Republic of Korea; Medical College of Wisconsin, United States of America

## Abstract

The adult mammalian heart has limited capability for self-repair after myocardial infarction. Therefore, therapeutic strategies that improve post-infarct cardiac function are critically needed. The small molecule ICG-001 modulates Wnt signaling and increased the expression of genes beneficial for cardiac regeneration in epicardial cells. Lineage tracing experiments, demonstrated the importance of β-catenin/p300 mediated transcription for epicardial progenitor contribution to the myocardium. Female rats given ICG-001 for 10 days post-occlusion significantly improved ejection fraction by 8.4%, compared to controls (*P*<0.05). Taken together, Wnt modulation via β-catenin/CBP inhibition offers a promising therapeutic strategy towards restoration of myocardial tissues and an enhancement of cardiac functions following infarction.

## Introduction

The adult myocardium has a limited ability to repair, and during the healing process after an ischemic injury, it is predominantly replaced with fibrotic tissue. Higher risk of fatal heart failure results from impaired contractile function due to fibrotic scarring. In adults, the number of resident stem/progenitor cells and their ability to efficiently restore functional heart tissue appears to be quite limited. Thus, the restoration of functional myocardium in the infarcted adult heart is an important goal.

Recent studies utilizing stem cell therapies for myocardial infarction (MI) have mainly focused on supplementing insufficient cardiac stem/progenitor cells with exogenous cells. Transplantations of embryonic stem cells into the infarcted heart for cardiac regeneration in animals [1] have been performed, but this therapeutic strategy has many concerns in the clinical setting. An alternative approach is to pharmacologically manipulate endogenous stem/progenitor cells to increase repair. Such an approach, which promotes regenerative signaling pathways that are generally suppressed in adult mammals, has many advantages over surgical transplantation of cells [2].

Modulation of the Wnt signaling pathway provides a potential pharmacologic target for regenerative signaling in damaged myocardial tissue. Wnt signaling is required to direct critical biological processes during cardiac development, and is important in both the proliferation and differentiation of various stem/progenitor cell populations [3], [4]. Nuclear β-catenin mediates the Wnt-dependent transcriptional activation of a range of Wnt responsive target genes. ICG-001 [5] is a small molecule Wnt signaling modulator that specifically blocks the interaction between β-catenin and one of its Kat3 coactivators, CREB binding protein (CBP). ICG-001, however, does not interfere with the highly homologous interaction between β-catenin and the coactivator E1A binding protein p300.

We have previously reported on the ability of ICG-001 to initiate a differentiation program in a variety of stem/progenitor cell populations [6]–[8]. Furthermore, we have previously reported that the small molecule IQ1, which inhibits the binding of p300 to β-catenin, selectively enhances the CBP/β-catenin interaction thus preventing the differentiation of stem/progenitor cells [9]. IQ-1 therefore serves as a useful tool for investigating the effects of differential β-catenin coactivator usage opposite to those induced by ICG-001.

Selective inhibition of β-catenin/CBP-mediated activation of transcription by ICG-001 initiates the differentiation of stem/progenitor cells that are required for regeneration of neurons and the hematopoietic system [6], [7], [10]. In addition, inhibition of Wnt signaling has proven to have beneficial effects on the infarcted myocardium [11]–[13] and facilitates cardiomyocyte differentiation of murine embryonic stem cells [14]. Furthermore, ICG-001 may potentially limit cardiac fibrosis following infarction; we have previously demonstrated that ICG-001 dramatically reduces fibrosis and promotes re-epithelialization in mouse models of idiopathic pulmonary fibrosis (IPF) [8] and renal fibrosis [15]. Accordingly, inhibition of the β-catenin/CBP interaction with ICG-001 represents a potential therapeutic approach to enhance the ability of endogenous stem/progenitor cells to differentiate and repair the myocardium post-infarction.

To test the hypothesis, we initially examined CBP/p300 differential coactivator usage by β-catenin *in vitro,* particularly in regards to effects on the epicardium, which has been demonstrated to play an important role in contributing to both cardiac development and regeneration [16], [17]. Finally, we examined if specific β-catenin/CBP inhibition using ICG-001 has a beneficial effect in a rat model of myocardial infarction.

## Results

### The Epicardium is a Source of Progenitor Cells and Paracrine Factors for Cardiac Regeneration

**Figure 1:**
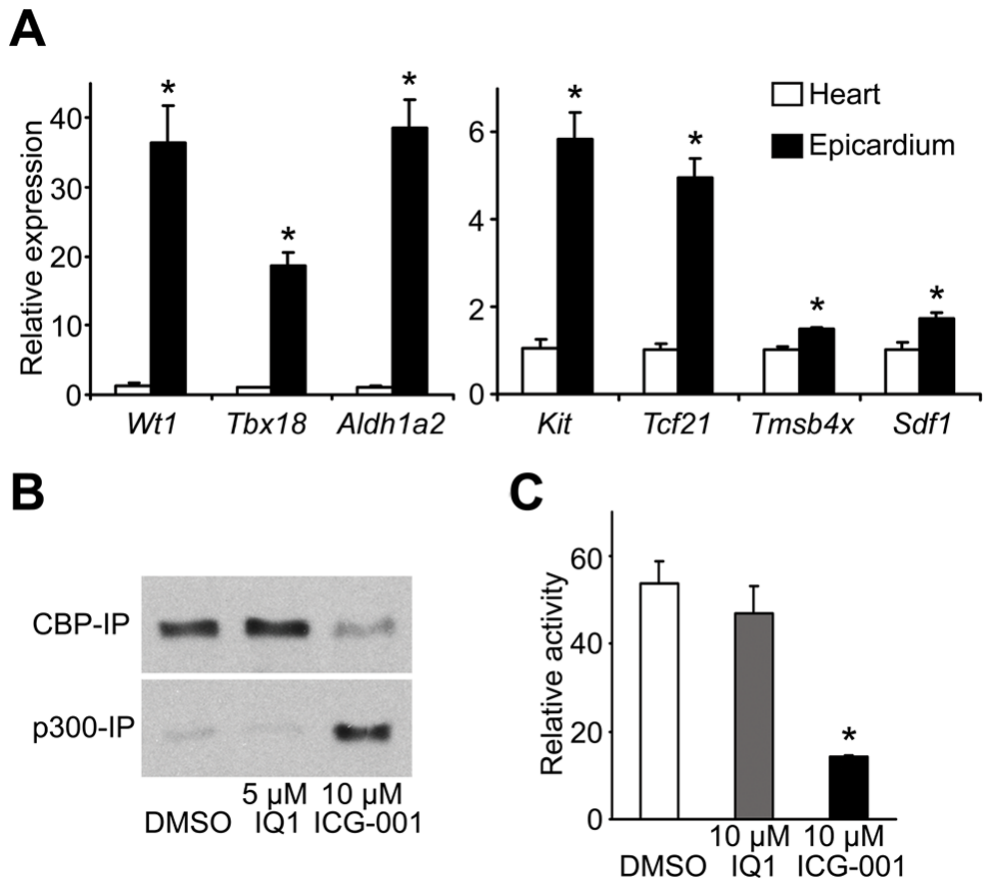
ICG-001 modulates Wnt signaling in rat epicardial mesothelial cells (EMCs). (A) Quantitative PCR analysis (qPCR) of mouse hearts and epicardium demonstrates that the epicardium expressed significantly higher levels of *Wt1*, *Tbx18*, *Aldh1a2*, *Ki*t, *Tcf21*, *Tmsb4x* and *Sdf1* than the remainder of the heart. *P<0.05. (B) β-catenin co-immunoprecipitation (co-IP) analysis shows that ICG-001 induced β-catenin coactivator switching from CBP to p300 in rat epicardial cells (EMCs). ICG-001 decreased β-catenin interaction with CBP (top panel) and increased the interaction with p300 (bottom panel) in rat EMCs. IQ1 slightly increased β-catenin interaction with CBP (top panel) and did not change the interaction with p300 (bottom panel). (C) Rat EMCs were transiently transfected with the TOPFlash reporter plasmid and assessed for luciferase activity. ICG-001 significantly decreased TOPFlash activity in rat EMCs. *P<0.05. Data are presented as mean ± s.e.m.

The epicardium consists of a single layer of mesothelial cells that attach to and spread over the myocardium during cardiac development [18]. In recent linage tracing studies using transgenic mice, epicardial cells have been suggested as a potential source for cardiomyocytes, endothelial and smooth muscle cells during cardiac development [16]. The epicardium also contributes essential cellular and paracrine factors during embryonic cardiac development and in principle may provide similar contributions post-infarction. To examine potential epicardium-derived contributing factors to cardiac regeneration, we compared epicardial specific expression of several genes reported to be important factors in cardiac regeneration, specifically in the epicardium compared with their expression in whole adult mouse hearts by quantitative PCR (qPCR). As anticipated, our results indicate that mouse epicardium expressed significantly higher levels of the epicardial cell markers, *Wt1, Tbx18, Aldh1a2* and *tcf21* [19] compared to total heart (Figure 1A). Additionally, the epicardium expressed significantly higher levels of a cardiac stem cell marker *Kit* [20], the epicardial growth factor *Tmsb4x* (*Thymosin β4*) [21] and a cardiac stem cell homing factor *Sdf1* [22] compared to total heart (Figure 1A). These results, which are consistent with recent data from other labs [23], [24], suggest that the epicardium may serve as a source for both stem/progenitor cells and paracrine factors required for cardiac regeneration.

### ICG-001 Modulates Wnt Signaling in Rat Epicardial Cells

Next, we examined the effect of modulating Wnt signaling *in vitro* in epicardial cells. To generate a transcriptionally active Wnt signaling complex, nuclear β-catenin recruits either the Kat3 coactivator CBP or its highly related homolog p300. Differential usage of the β-catenin/CBP or β-catenin/p300 complexes leads to activation of different subsets of Wnt target genes [25]. We have previously demonstrated that ICG-001 reduces the β-catenin/CBP interaction and thereby increases the β-catenin/p300 interaction in a variety of cell types [5], [9]. In contrast, IQ1, a specific small molecule antagonist of the β-catenin/p300 interaction has demonstrated the ability to maintain potency in stem/progenitor cell populations, including cardiovascular progenitors, by increasing the β-catenin/CBP interaction at the expense of the β-catenin/p300 interaction [9], [26]. In the event, we treated rat EMCs, an epicardial mesothelial cell line derived from an adult rat heart explant [27], with either ICG-001 or IQ1 and performed co-immunoprecipitation (co-IP) assays. Cells were treated with DMSO, ICG-001 or IQ1 for 24 hours. In the DMSO control treated cells, essentially all of the β-catenin was associated with CBP (Figure 1B, DMSO). Treatment with IQ1 had minimal effects on β-catenin coactivator usage (Figure 1B, IQ1). However, as anticipated, treatment with ICG-001 decreased the β-catenin/CBP interaction, while concomitantly increasing the β-catenin/p300 interaction (Figure 1B, ICG-001).

Next, we examined the effects of ICG-001 on the canonical Wnt signaling cascade in rat epicardial cells using the TOPFlash reporter assay [28]. TOPFlash luciferase is activated by β-catenin binding to TCF/LEF binding sites in the construct. Rat EMCs transiently transfected with the TOPFlash reporter plasmid displayed significant TOPFlash luciferase activity (Figure 1C). The specific β-catenin/CBP antagonist ICG-001 significantly down-regulated TOPFlash luciferase activity, whereas IQ1, the specific β-catenin/p300 antagonist did not affect TOPFlash activity in these cells (Figure 1C). Taken together, these results demonstrated that ICG-001 can modulate Wnt signaling by selectively antagonizing the β-catenin/CBP interaction in a rat epicardial cell line.

### ICG-001 Modulates Gene Expression in Rat EMCs

**Figure 2:**
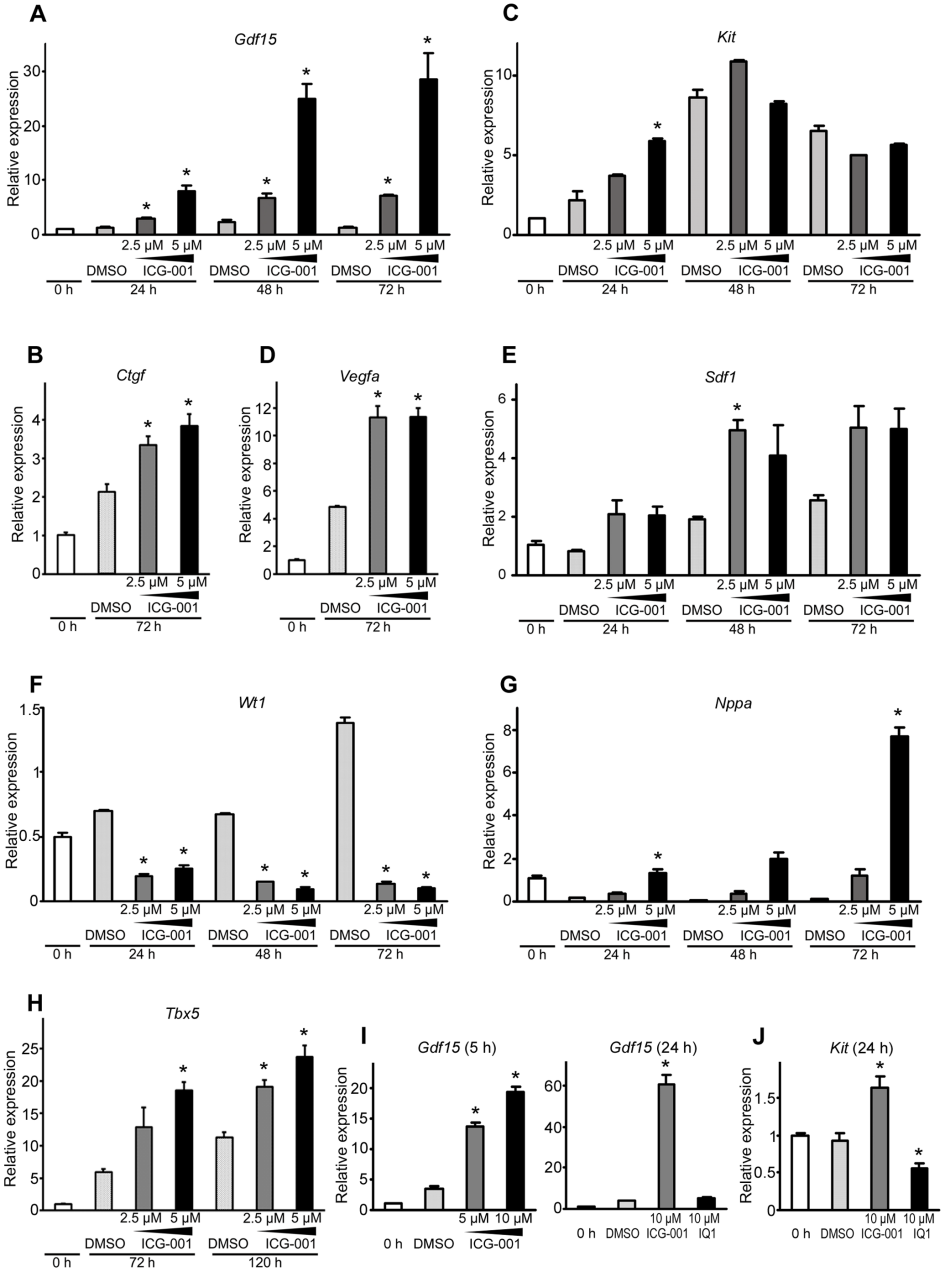
qPCR analysis of rat EMCs and mouse heart explants treated with ICG-001. (A-E) ICG-001 significantly increased mRNA expression of *Gdf15* (2.5 µM and 5 µM at 24, 48 and 72 h), *Ctgf* (2.5 µM and 5 µM at 72 h), *Kit* (5 µM at 24 h), *Vegfa* (2.5 µM and 5 µM at 72 h) and *Sdf1* (2.5 µM at 48 h) in rat EMCs. *P<0.05. (F) ICG-001 significantly decreased the expression of *Wt1* in rat EMCs. *P<0.05. (G-H) ICG-001 significantly increased the expression of *Nppa* and *Tbx5* in rat EMCs. *P<0.05. (I) qPCR analysis for *Gdf15* expression in mouse embryonic heart explants. ICG-001 significantly increased the expression of *Gdf15* at both 5 hours and 24 hours. *P<0.05 (J) qPCR analysis of *Kit* mRNA expression in mouse embryonic heart explants. ICG-001 significantly increased the expression of *Kit* at 24 hours. IQ1 significantly decreased the expression of *Kit* at 24 hours. *P<0.05. Data are presented as mean ± s.e.m.

We next examined if ICG-001 modulates the expression of genes, which may be involved in the promotion of cardiac regeneration, in rat EMCs by qPCR. Rat EMCs treated with ICG-001 significantly increased the expression of *Gdf15*, a growth factor previously shown to protect against cardiac rupture [29], (Figure 2A), *Ctgf* (*Ccn2*) a Wnt target gene known to regulate angiogenesis [30] and attenuate cardiac remodeling [31] (Figure 2B), the cardiac stem cell marker *Kit* (Figure 2C), the angiogenic growth factor *Vegfa* [32] (Figure 2D) and the cardiac stem cell homing factor chemokine *Sdf1* [22] (Figure 2E). We also found that ICG-001 significantly decreased the expression of the epicardial lineage marker *Wt1* (Figure 2F) and increased the expression of a cardiomyocyte-specific marker *Nppa* [33], particularly at 72 h (Figure 2G) and the early cardiac transcription factor *Tbx5* [34] (Figure 2H). Decreased *Wt1* expression with a concomitant increase in *Nppa* and *Tbx5* expression suggested that ICG-001 promoted the differentiation of epicardial progenitors thereby contributing to myocardial regeneration potentially via multiple mechanisms (i.e. cardiomyocyte generation and angiogenesis). As such, our qPCR results suggest that ICG-001 can modulate genes that are associated with a cardiac regeneration gene cassette in rat EMCs.

### ICG-001 Increases *Gdf15* and *Kit* in Mouse Heart Organ Culture

ICG-001 increased the expression of *Gdf15* and *Kit* in rat EMCs. To verify this observation at the whole organ level, we utilized an *ex vivo* mouse heart organ culture system. Whole mouse embryonic hearts were cultured in media containing either DMSO or ICG-001. After treatment, the ventricles were collected from the explants for qPCR analysis. We found that ICG-001 treatment significantly increased the expression of *Gdf15* in the ventricles of explants at both 5 and 24 h after treatment (Figure 2I). ICG-001 treatment also significantly increased the expression of *Kit* at 24 h (Figure 2J). Our observation that ICG-001 increases the expression of *Gdf15* and *Kit* in heart explants suggested that ICG-001 could have potential benefits during cardiac regeneration after injury.

### ICG-001 Promotes Epithelial to Mesenchymal Transition (EMT) in Epicardial Cells

**Figure 3:**
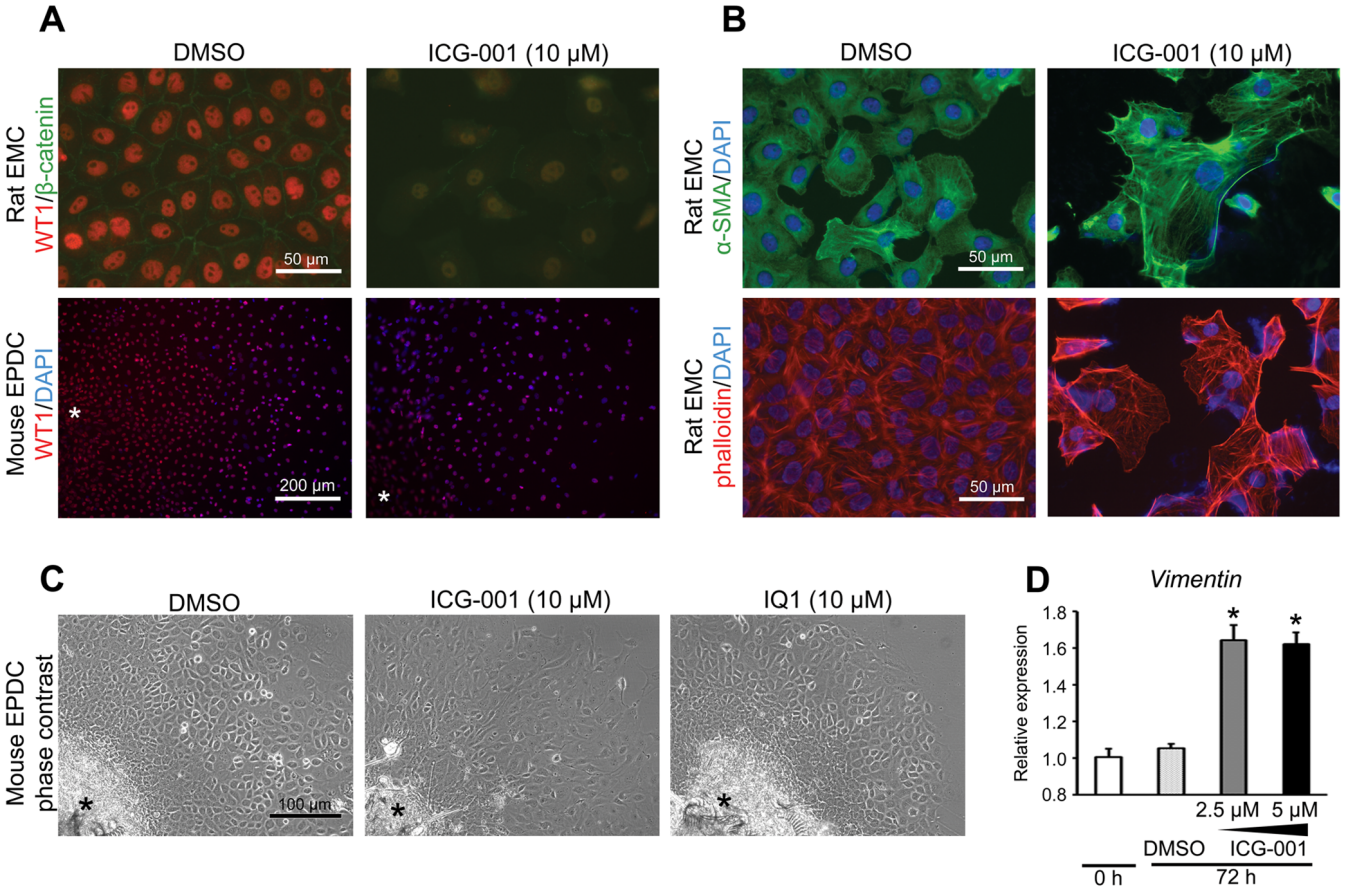
Immunocytochemical analysis of epicardial cells treated with ICG-001. (A) ICG-001 decreased nuclear WT1 protein expression, an epicardial cell marker, by immunocytochemistry, both in rat EMCs and mouse epicardium-derived cells (mouse EPDCs). Fluorescence colors: red; WT1, green; β-catenin, blue; DAPI/nuclear stain. *; Original epicardial explant. (B) ICG-001 increased α-SMA smooth muscle actin filaments in rat EMCs. Fluorescence colors: green; α-SMA, red; phalloidin/F-actin filament, blue; DAPI/nuclear stain. (C) ICG-001 decreased the cobblestone patterning of mouse EPDCs by phase contrast microscopy. *; Original epicardial explant. (D) ICG-001 significantly increased the expression of *Vimentin* by qPCR in rat EMCs. *P<0.05. Data are presented as mean ± s.e.m.

ICG-001 treatment decreased the mRNA expression of the epicardial cell marker *Wt1* in rat EMCs as indicated by qPCR (Figure 2F). To confirm this result at the protein level, we examined WT1 expression by immunocytochemistry in rat EMCs and mouse primary epicardium-derived cells (EPDCs). Consistent with the qPCR results, ICG-001 decreased the levels of WT1 protein in both rat EMCs and mouse epicardium-derived cells (Figure 3A). Concomitant with the down-regulation of WT1, ICG-001 treated rat EMCs underwent a dramatic morphology change, from a neatly arranged cobble-stone “epithelial-like” morphology to a more mesenchymal phenotype, as judged additionally by α-SMA and F-actin (phalloidin) staining (Figure 3B). Similarly, epicardium-derived cells treated in culture with ICG-001 also underwent a morphology change, resulting in diminished cell-cell contact, as compared to either DMSO control or cells treated with the β-catenin/p300 antagonist IQ1 (Figure 3C). ICG-001 also significantly increased the expression of the EMT maker, *Vimentin* by qPCR at 72 hours in rat EMCs (Figure 3D). For an epicardial progenitor to contribute as a cell source for myocardial regeneration, the epicardial cell must initially undergo an epithelial to mesenchymal transition (EMT); i.e. the epicardial cells must first lose their “epithelial-like” characteristics and migrate into the myocardium. β-catenin has been shown to be essential for normal epicardial EMT [35]. Accordingly, our results imply that ICG-001, by specifically interfering with β-catenin/CBP transcription, promotes EMT and increases the differentiation of epicardial progenitor cells, thereby contributing to the regeneration process after myocardial infarction.

### Differential β-catenin Coactivator Usage is Critical during Cardiogenesis

**Figure 4:**
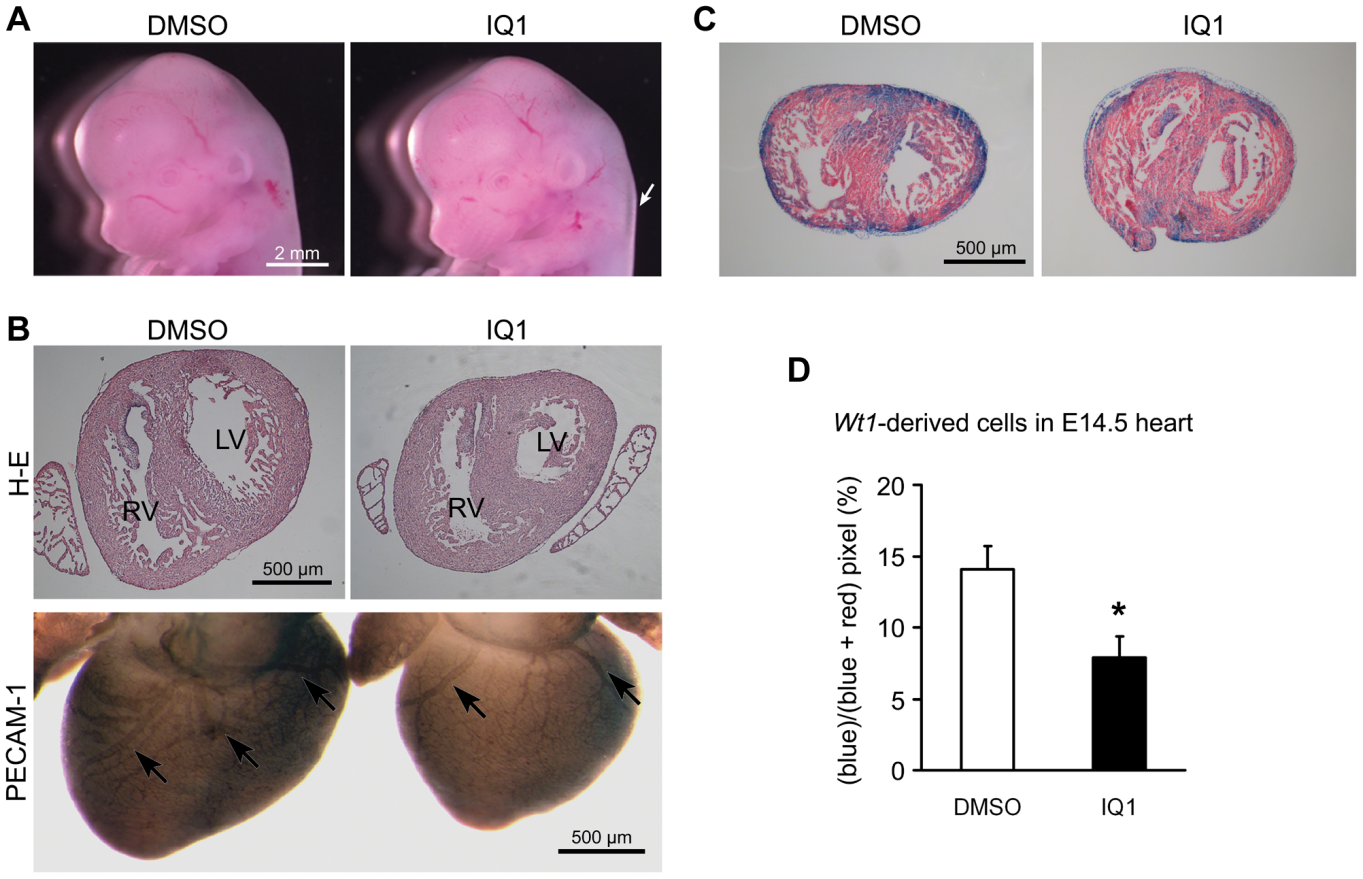
Effects of IQ1 on mouse embryonic hearts. (A) Mouse embryos were exposed to IQ1 from E12.5 to E13.5 *in utero*. Edematous skin is observed in IQ1 treated embryos (arrow). (B) Embryos were exposed to IQ1 from E12.5 to E16.5. IQ1 reduced the size of the embryonic hearts, size of ventricular spaces and number of trabeculae in the ventricles in transverse H-E sections (top). Whole mount PECAM-1 immunostaining shows the coronary vascular network from a dorsal view of the hearts (arrows, bottom). IQ1 decreased the number of blood vessels. LV; left ventricle, RV; right ventricle. (C) Mouse embryos were exposed to IQ1 from E12.5 to E14.5 *in utero*. β-galactosidase activity (blue) shows *Wt1*-derived cells in transverse heart sections. Nuclei were counterstained with Nuclear Fast Red. (D) *Wt1* **-**derived cells were quantified by blue pixels. IQ1 treatment decreased *Wt1*-derived cells in mouse embryonic hearts significantly. *P = 0.0197. Data are presented as mean ± s.e.m.

During development, epicardial cells migrate from the proepicardium and spread over the surface of the heart. A subset of epicardial cells subsequently migrate into the subjacent myocardium, and differentiate into smooth muscle and endothelial cells [36], [37]. To investigate the importance of the β-catenin/p300 interaction, which is increased by ICG-001 treatment, we used the small molecule IQ1 to perform a pharmacologic “loss-of-function” analysis to selectively antagonize the β-catenin/p300 interaction during mouse cardiac development. We anticipated that blocking the β-catenin/p300 interaction during cardiac development would prevent epicardial EMT and subsequent differentiation. Timed pregnant mice were treated with IQ1 (0.5 M, 0.25 µl/kg/day) from E12.5 until E16.5 to expose the embryos to IQ1 *in utero*. Blocking the β-catenin/p300 interaction with IQ1 negatively impacted cardiac development. Embryos treated with IQ1 for only one day showed edema, indicative of cardiac malfunction, even though there was no general growth delay (Figure 4A). After 4 days of IQ1 treatment, the embryonic hearts were smaller and had smaller ventricular spaces (Figure 4B). IQ1 also decreased the number of coronary blood vessels as judged by whole mount staining of the endothelial marker, PECAM-1 (Figure 4B, bottom). These results suggest that β-catenin/p300-driven transcription is critical for normal cardiac development. Next, we analyzed the effect of antagonizing the β-catenin/p300 interaction in embryonic hearts using epicardial lineage tracing of *Wt1*-derived cells. *Wt1*-derived epicardial progenitors were genetically labeled with β-galactosidase via crossing *Wt1-Cre* mice and *Rosa26R* mice as previously described [16]. Embryonic mice were treated in utero, as described above, with IQ1 from E12.5 to E14.5. *Wt1*-derived blue-labeled cells were found in both the epicardium and myocardium in transverse sections of the heart (Figure 4C). Quantification of the area of *Wt1*-derived cells as blue pixels and the area of non-*Wt1*-derived cells as red pixels was performed. IQ1 significantly decreased the area of the *Wt1*-derived cells (Figure 4D). This result further indicates that the β-catenin/p300 interaction plays an important role in the contribution of *Wt1* positive epicardial progenitors to embryonic heart development.

### ICG-001 Improves Cardiac Contractile Function in a Rat Myocardial Infarction Model

**Figure 5:**
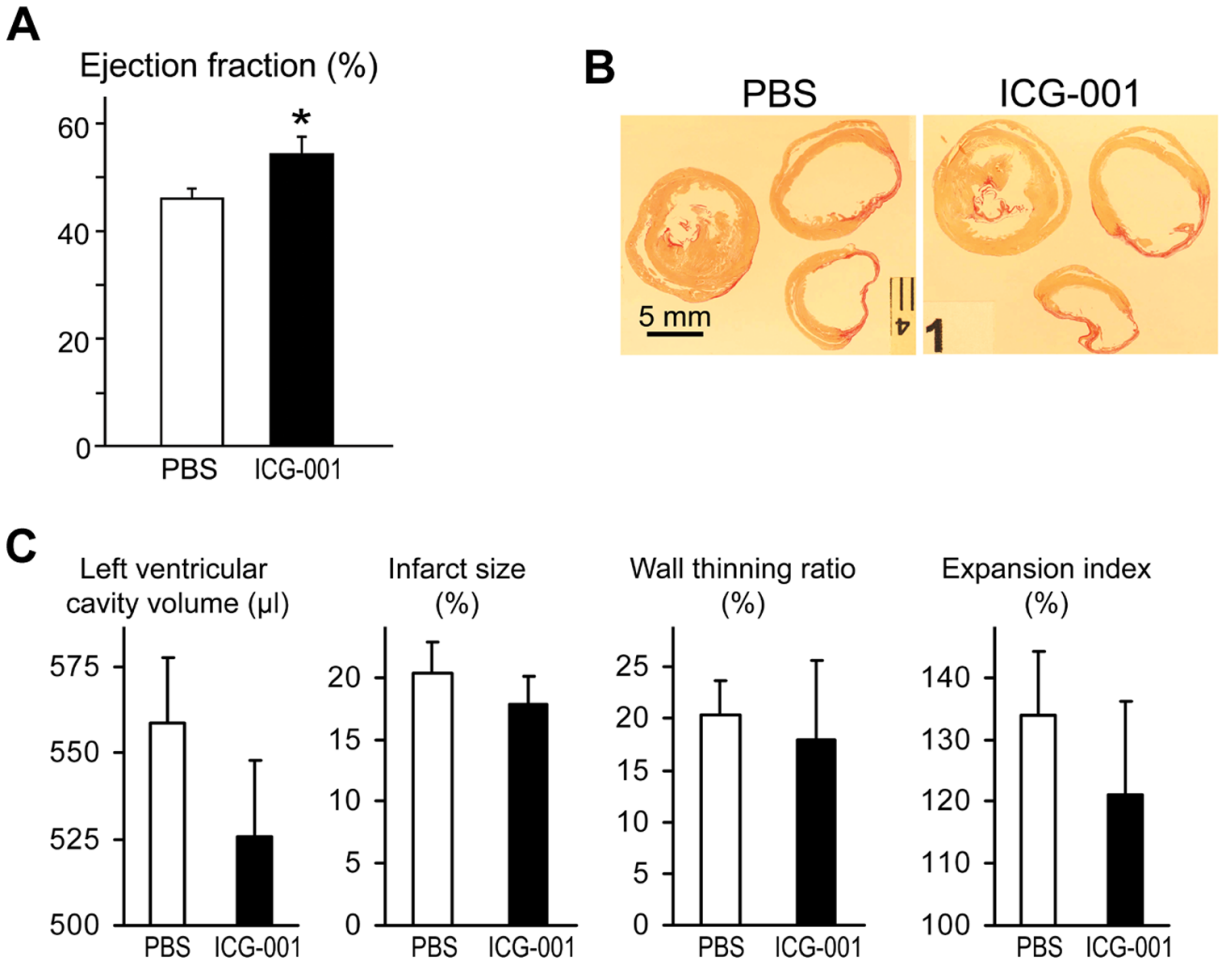
ICG-001 effects on rat myocardial infarction (MI). (A) ICG-001 improved left ventricular ejection fraction at 4 weeks post-coronary artery occlusion. Data are presented as mean ± s.e.m. *P = 0.031 by two-tailed student’s t-test (PBS *n = *11, ICG-001 *n = *9). (B) Representative transverse sections of hearts at 4 weeks post-surgery. Picrosirius red staining shows a thin collagen rich scar after myocardial infarction. Yellow; muscle, red; collagen. No visible difference in collagen deposition was observed. (C) Left ventricular histomorphometry at 4 weeks post-surgery. ICG-001 insignificantly improved left ventricular cavity volume, infarct size, wall thinning ratio and expansion index. However, no statistical significance was found in this experimental size (*P*>0.05). Data are presented as mean ± s.e.m.

Based on the results above, we hypothesized that ICG-001 could have a beneficial therapeutic effect post-myocardial infarction via modulation of Wnt signaling. To investigate the therapeutic effects of ICG-001 in the process of repair and regeneration of cardiac function, we utilized a female rat model of myocardial infarction [38]. In this model of myocardial infarction, we have typically observed severe infarct expansion, scar thinning, LV dilation and remodeling and the development of heart failure within 4 weeks post-surgery. There is also a marked fall in ejection fraction, baseline being around 80%, which progressively worsens over time [39]. In the event, the left coronary artery of female Sprague-Dawley rats was permanently occluded via surgery to induce regional ischemic injury to the left ventricle. ICG-001 was administered to the rats subcutaneously (50 mg/kg/day) beginning on the day of surgery for 10 days. Four weeks after surgery (20 days after the last ICG-001 treatment), left ventricular ejection fraction was assessed by angiography as an indicator of cardiac contractile function. We found that ICG-001 significantly improved ejection fraction by 8.4% from 46.2±1.7% to 54.6±3.4% (*P*<0.05) (Figure 5A). This data demonstrates that ICG-001 significantly improved cardiac contractile function after myocardial infarction in the rats.

We next analyzed the hearts by histomorphometry to investigate the effects of ICG-001 on cardiac morphology. Transverse sections of the hearts at 4 weeks post-infarction showed collagenous transmural scars, thinning of the ventricular wall and expansion of the ventricular wall at the infarct site in both the ICG-001 and PBS groups (Figure 5B). The thinning and expansion of the ventricular wall, a result of degeneration of the myocardium, collectively impairs cardiac contractile function. The ICG-001 group showed a trend towards preserving cardiac morphology in regards to left ventricular cavity volume, infarct size, wall thinning ratio, and expansion index (Figure 5C). However, these changes failed to reach statistical significance.

### Mechanistic Investigations on the Beneficial Effects of ICG-001 Post-Myocardial Infarction

**Figure 6:**
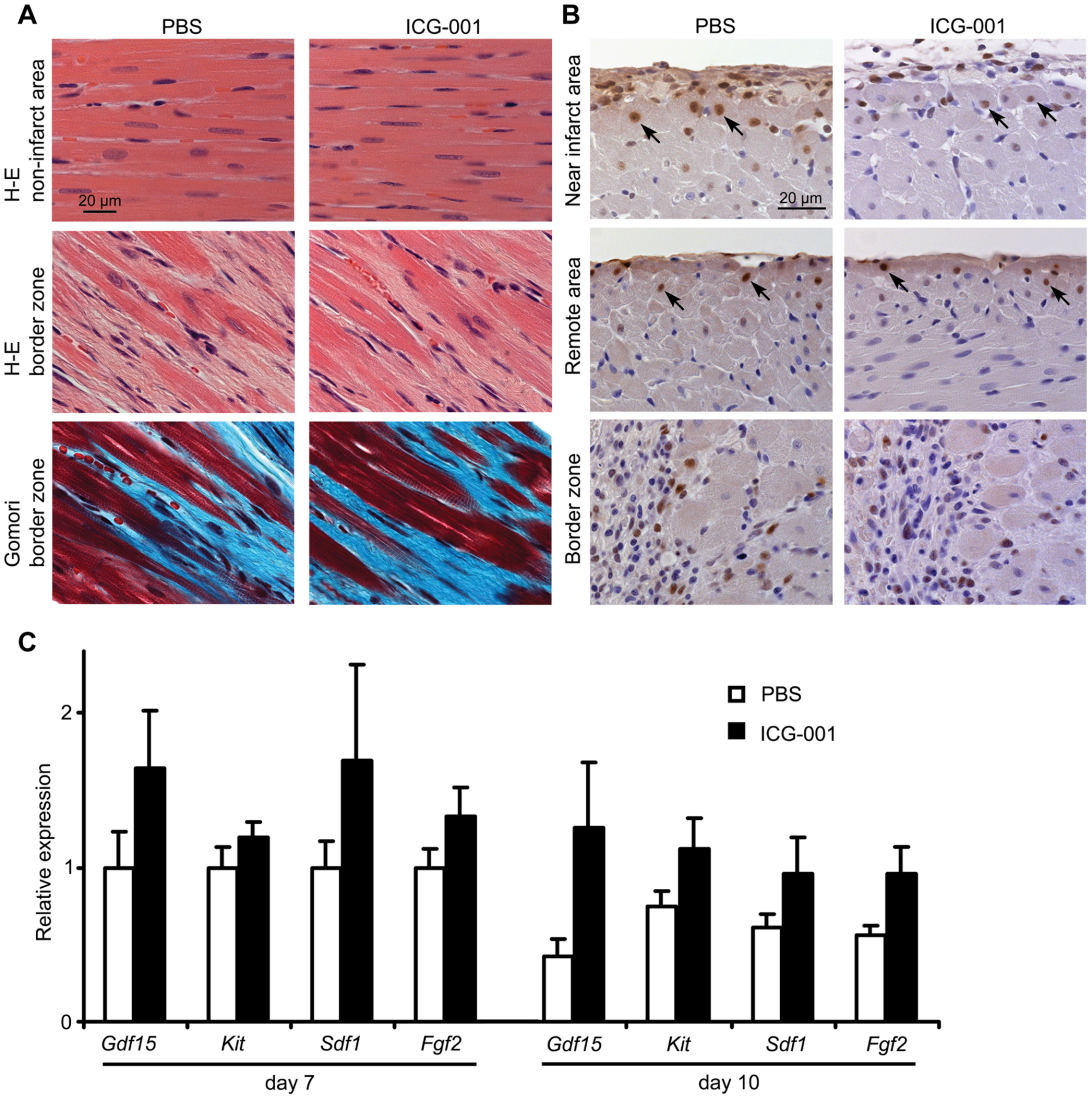
Histology and gene expression analysis of ICG-001 treated rats from the myocardial infarction model. (A) Photomicrographs of transverse sections of the hearts at 10 days post-surgery. ICG-001 did not histologically change the hearts at the non-infarcted areas and the border zone by H-E staining (top and middle panel) and Gomori’s trichrome staining (bottom panel). Red; muscle, blue green; collagen, dark purple; hematoxylin/nuclear stain. Contribution of epicardium to cardiac regeneration. (B) Continuous administration of BrdU labeled replicating cells in the adult rat hearts at 7 days post-coronary occlusion. Brown; BrdU positive staining, dark purple; hematoxylin/nuclear stain. BrdU positive nuclei were found in cardiomyocytes near the epicardium (top and middle panel). BrdU positive nuclei occupy the center of the cross-sectioned cardiomyocytes (arrows). BrdU positive nuclei were also found in granulation tissue at the border zone but not in cardiomyocytes (bottom panel). (C) qPCR analysis of hearts at 7 and 10 days post-coronary occlusion in adult rats. ICG-001 showed a trend toward increased expression of *Gdf15*, *Kit, Sdf1* and *Fgf2* at both day7 and day10, but statistical significance was not reached (*P*>0.05). Data are presented as mean ± s.e.m.

To investigate the mechanism(s) by which ICG-001 improves cardiac contractile function, we treated an additional set of rats with or without ICG-001 after undergoing left coronary artery occlusion. The rats were euthanized at 7 and 10 days post-surgery. Transverse sections of the hearts at 10 days post-surgery were examined after H-E and Gomori’s trichrome staining (Figure 6A). We observed few hypertrophic cardiomyocytes along with an irregular arrangement of myofibrils at the border zones. We also measured the area of fibrotic tissue by histomorphometry, and found that ICG-001 did not significantly affect the amount of fibrotic tissue (Figure S1). In summary, there were no major differences in the pathologic findings between the two groups.

To investigate potential proliferative progenitor populations in the infarcted heart, we performed a BrdU (bromodeoxyuridine) incorporation experiment. We administered BrdU to the rats in their drinking water, immediately post-surgery until the day of euthanasia, to provide continuous exposure. We observed BrdU positive cells primarily in the infarcted epicardium and subepicardium, and in granulation tissue at the infarct border zone (Figure 6B). BrdU positive cardiomyocytes were distinguishable from other BrdU positive cells by their distinct morphology consisting of a centrally located nucleus in cross-sectioned muscle fibers. BrdU positive cardiomyocytes were found in both the PBS and ICG-001 treated hearts in the region adjacent to the epicardium (Figure 6B, arrows), but rarely in the border zone (Figure 6B, border zone). These observations are consistent with either the replication of cardiomyocytes post-infarction or the proliferation of progenitors that subsequently differentiated into cardiomyocytes. This process occurred more frequently adjacent to the epicardium than at the border zone. Despite these observations, we could not statistically evaluate the differences in the number of BrdU positive cardiomyocytes between PBS and ICG-001 treated hearts due to the overall low number in our samples. However, the results of the BrdU incorporation experiment were consistent with an epicardial contribution to the regeneration of cardiomyocytes in adult rat hearts.

We next examined the expression of various genes implicated in cardiac regeneration in the infarcted rat hearts. These genes included *Gdf15, Kit* and *Sdf1*, which we had earlier shown to be modulated by ICG-001 in rat EMCs (Figures 2A, 2C and 2E) and mouse embryonic heart (Figures 2I and 2J). Although the calculated differences were not statistically significant within our experimental group size, there was a clear trend towards increased *Gdf15*, *Kit*, *Sdf1* and *Fgf2* expression with ICG-001 treatment at both day 7 and day 10 (Figure 6C), consistent with our *in vitro* results. These results imply that genes shown *in vitro* to be regulated by ICG-001, including *Gdf15*, *Kit*, *Sdf1* and *Fgf2,* are genes potentially regulated by ICG-001, are apparently similarly regulated *in vivo* in infarcted rat hearts. However, further larger scale studies and protein expression will be required to assess the significance of these results.

## Discussion

Our results collectively indicate that β-catenin/CBP signaling modulation may offer an attractive pharmacologic strategy to treat myocardial infarction (MI) via enhancing an endogenous repair process. ICG-001 improved cardiac function significantly in a female rat chronic myocardial infarction model *in vivo*, as reflected in the significantly increased ventricular ejection fraction as judged by left ventricular angiogram [40], a standard technique for assessing LV ejection fraction that remains the gold standard for assessing LV function in humans at the time of cardiac catheterization [39]. We have previously shown that administration of 5 mg/kg/day continuous infusion in a bleomycin induced pulmonary fibrosis model in mice was both therapeutically efficacious and reduced Wnt signaling as judged by reduction of X-Gal staining in the lungs in the Bat-Gal transgenic reporter mice utilized in these studies [8]. As ICG-001 was given i.p. bolus in this set of experiments, we used a higher dose (50 mg/kg/day) to insure better coverage for an extended period of time as ICG-001 has a limited half-life in rodents. Our result is consistent with previous findings that intervention in Wnt signaling may be beneficial for the treatment of myocardial infarction. Genetic modulations of Wnt signaling [41], [42], administration of Wnt biomolecules [13], [43], or administration of the Wnt signaling inhibitor Pyrvinium [44] improved cardiac remodeling after myocardial infarction [45]. However, the therapeutic value of these interventions is severely limited due to feasibility and toxicity. In contrast, PRI-724, a second generation β-catenin/CBP antagonist has recently completed Phase Ia clinical evaluation and has proven to be extremely safe in humans [46] and therefore in principle could be readily administered therapeutically post-myocardial infarction.

Biological mechanisms potentially contributing to the beneficial effects of targeting Wnt β-catenin/CBP signaling after myocardial infarction include inhibition of fibrosis, apoptosis and hypertrophy of the myocardium and activation of regenerative cells [45]. In this study, we demonstrate that the effects of Wnt modulation of the epicardium, a cardiac regenerative cell source, may play a significant role in the beneficial effects of ICG-001 administration post-myocardial infarction. The epicardium is a rich source for vascular endothelial and progenitor cells [47]–[49]. The epicardium has recently been suggested to contribute to cardiac regeneration based on lineage tracing studies in mice [16], [50] and heart regeneration studies in zebrafish [51], [52]. Interestingly, ICG-001 significantly increased the expression of *Gdf15, Ctgf, Kit, Vegfa, Sdf1*, *Nppa* and *Tbx5* in a rat epicardial cell line (rat EMC) (Figures 2A, 2B, 2C, 2D, 2E, 2G and 2H). GDF15, a distant member of the TGF-β family has been demonstrated to offer protection against cardiac rupture [29]. The growth factor *Ctgf* (*Ccn2*), a known Wnt target, has previously been shown to both regulate angiogenesis [30] and attenuate cardiac remodeling [31]. c-KIT, which is encoded by *Kit*, is a cardiac stem/progenitor marker [20], *Vegfa* is a potent angiogenic factor [32], SDF1 is involved in cardiac stem cell homing [22], *Nppa* is a cardiomyocyte differentiation marker [33] and *Tbx5* is an early cardiac transcription factor [34]. These results suggest that ICG-001 potentially enhances cardiac regeneration by multiple mechanisms including both increased activation and subsequent differentiation of stem/progenitor cells, enhanced angiogenesis as well as increased expression of cytokines and chemokines in the epicardium and myocardium that aid in cardiac regeneration. Interestingly, in mouse models of cardiac hypertrophy and myocardial infarction, GDF15 acts as a novel autocrine/endocrine factor that antagonizes the hypertrophic response and loss of ventricular performance, thereby preventing myocardial apoptosis and limiting infarct size [53]. ICG-001 induced expression of GDF15 may at least partially contribute to the beneficial effects of ICG-001 treatment on the myocardial repair process. However, it should be noted that high levels of GDF15 were recently associated with a poorer prognosis in patients with acute myocardial infarction [54]. The reason for these apparently conflicting results is not clear although they could be related to temporal expression.

Enhancement of epithelial to mesenchymal transition (EMT) of epicardial progenitor cells, invasion of the myocardium and subsequent differentiation, is another possible mechanism whereby ICG-001 could contribute to cardiac regeneration. During cardiac development and regeneration, epicardial cells undergo EMT and delaminate, migrate into the subjacent myocardium and potentially differentiate into cardiomyocytes, fibroblasts, smooth muscle cells or endothelial cells [24], [55]. The expression of the epicardial marker WT1 decreases as epicardial-derived cells differentiate [56]. Indicative of the ability of ICG-001 to promote the EMT/differentiation process, we showed that ICG-001 significantly downregulates WT1 at both the mRNA and protein levels (Figures 2F and 3A). ICG-001 also changed epithelial into mesenchymal morphology in mouse epicardium-derived cells (Figures 3B and 3C). ICG-001 increased the expression of the EMT maker *Vimentin* [57] by qPCR significantly in rat EMCs (Figure 3D). These data imply that ICG-001 affects an increase in migratory capacity and the subsequent promotion of migrated epicardial-derived cells into myocardium or endothelium. Thus, ICG-001 by recapitulating processes involved in embryonic cardiac development may enhance the migration and differentiation of proliferating epicardial cells after infarct, thereby further contributing to the cardiac repair process.

To further support our contention that selectively antagonizing the β-catenin/CBP interaction is beneficial for cardiac regeneration, we demonstrate a negative impact of increasing the β-catenin/CBP interaction in mouse cardiac development. IQ1 is a specific small molecule inhibitor that enhances the β-catenin/CBP interaction at the expense of the β-catenin/p300 interaction. IQ1 decreased the contribution of the epicardium to myocardial development as judged by lineage tracing of *Wt1*-derived cells contributing to the embryonic heart (Figure 4D). This result indicates that the β-catenin/p300 interaction plays an important role in epicardial cell migration and differentiation during embryonic heart development. Interestingly, a heterozygous p300 knock-in model, in which the histone acetyltransferase domain was mutated [58], demonstrated multiple cardiac malformations including reduction of myocardial thickening, particularly in the ventricular wall, during development. This was not observed with the corresponding CBP knock-in. These investigators also noted a clear reduction in subepicardial mesenchymal cells (SEMCs) and decreased delamination of SEMC into the myocardium, phenotypically similar to the embryonic mouse hearts treated with the p300/β-catenin antagonist IQ1. We postulate that the post-infarct repair process in the adult, attempts to recapitulate this developmental process.

Although ICG-001 treatment consistently increased the expression of *Gdf15*, *Kit* and *Sdf1* at both day7 and day10 in rat hearts after myocardial infarction, we did not observe statistically significant changes (Figure 6C). This could be a result of the early time points (7 and 10 days post-infarct) examined relative to the time point examined for cardiac function (30 days post-infarct) and the apparently relatively protracted (more than 2 months) activation of the epicardium post-infarction (at least in rats) [59]. Alternatively, this could be due to a relatively small percentage of progenitor cells involved in the repair process compared to the overall ventricular mass or due to the large inter-animal variation and the limited number of rats per experimental group. Larger studies and optimization of the dosing regimen will be required to address these questions.

We cannot rule out other potential mechanisms that contribute to the overall positive effects that had been observed with ICG-001 treatment. Wnt signaling is a major regulatory signaling pathway of cardiac hypertrophy and fibrosis [60]. However, we did not observe significant changes by ICG-001 histologically in terms of hypertrophy and fibrosis at 4 weeks post-coronary artery occlusion. ICG-001 did not significantly affect LV wall thickness as judged by histomorphometry (Figure S2). We also did not observe significant changes in the expression of the cardiac hypertrophy marker genes *MCIP1*, *Nppa,* and *Nhe1* in rats treated with ICG-001 10 days post-coronary artery occlusion (Figure S3). From these observations, we believe that the cardiac hypertrophic response and scar stabilization were not the major factors contributing to the significant improvement in ejection fraction that we observed. However, we have previously demonstrated that ICG-001 decreases fibrosis and increases re-epithelialization in mouse models of idiopathic pulmonary fibrosis [8] and renal fibrosis [15]. ICG-001 also decreased the transcription of the TGF-β pro-fibrotic target gene PAI-1 [15]. PAI-1 has been previously demonstrated to have a profibrotic effect in the infarcted heart [61]. Therefore, ICG-001 by limiting fibrosis or possibly by promoting differentiation of cardiac fibroblasts or myofibroblasts to myocardium after myocardial infarction could also contribute to the positive effects seen in this model.

## Conclusion

We now demonstrate that the small molecule ICG-001 specifically antagonizes the β-catenin/CBP interaction in epicardial cells, a potential source of stem/progenitor cells for cardiac regeneration after myocardial infarction. In epicardial cells *in vitro* and *ex vivo,* ICG-001 activated the transcription of target genes beneficial to the cardiac regeneration process. Lineage tracing experiments during cardiac development demonstrated the importance of β-catenin/p300 mediated transcription to epicardial EMT and subsequent differentiation and contribution to the myocardium. Furthermore, β-catenin/CBP antagonism improved cardiac function in a female rat model of myocardial infarction. We anticipate that selective β-catenin/CBP antagonists may provide a pharmacologic strategy to improve outcome after myocardial infarction.

## Materials and Methods

### Rat Myocardial Infarction Model

All studies were approved by the Good Samaritan Hospital Animal Care and Use Committee and were conducted in accordance to the guide for Care and Use of Laboratory Animals (NIH publication 85-23, revised 1996). Good Samaritan Hospital research facility is fully accredited by USDA and AAALAC. Female Sprague-Dawley rats (200–250 g) were used for this study. On the day of surgery, rats were weighed and anesthetized with Ketamine/Xylazine (75 mg/kg Ketamine; 5 mg/kg Xylazine; i.p.). Animals were intubated and mechanically ventilated (60 cycles/min, 1 ml/100 g). A left thoracotomy was carried out. The heart was exposed through the fourth intercostal space. A silk suture (4-0) was placed around the proximal left coronary artery within 5 mm of its take-off from the aorta. The coronary artery was occluded by ligating the silk suture. The chest was closed, and the skin was stapled. The rats were under post-operative care for 3 days. Indications of pain and discomfort, appearance, activity, food and water intake and wound healing were monitored. Buprenorphine was injected twice daily during this period. The rats were randomly assigned into either an ICG-001 group or a phosphate-buffered saline (PBS) control group. The animals in the ICG-001 group received ICG-001 subcutaneously (25 mg/kg, twice daily) from the day of surgery for 10 days. Rats in the PBS group received PBS. Four weeks after the surgery, changes in cardiac contractile function were studied. Under full anesthesia (ketamine and xylazine cocktail, as described above), the rats were euthanized by a bolus injection of potassium chloride through the jugular vein. For post-mortem volume and histological analyses, the hearts were removed and formalin-fixed. Twenty animals were utilized for the assessments of cardiac function, post-mortem volume analysis, and histomorphometry (ICG-001 *n* = 9; PBS *n* = 11). BrdU and qPCR analysis were performed in 19 rats at 7 and 10 days of coronary occlusion (day 7: ICG-001 *n* = 6, PBS *n* = 6; day 10: ICG-001 *n = *4, PBS *n* = 3). Forty-nine animals were initially assigned to either ICG-001 or PBS control. Eight animals died either during surgery or within the first 24 hrs. after surgery. Two rats were excluded from data analysis due to failed infarction. Details of the BrdU assay are found in Text S1.

### Measurements of Cardiac Contractile Function

Ejection fraction was measured angiographically 4 weeks after coronary occlusion. This method has been adapted in various studies for the assessment of myocardial contractile function in infarcted hearts [40], [62], [63]. Briefly, rats were anesthetized with ketamine and xylazine (75 mg/kg Ketamine; 5 mg/kg Xylazine; i.p.). The right jugular vein was cannulated and non-ionic contrast agent (1 ml) was injected through the jugular venous catheter [40]. The edge of the ventricular cavity was well defined by the contrast. Video images were acquired with the XiScan 100 C-arm x-ray system (XiTec, Inc; 3-inch field of view, anterior-posterior (AP) and lateral projections) and recorded on half-inch super-VHS videotape at 30 frames per second under constant fluoroscopy. End-diastolic and end-systolic frames were selected by visually scanning for the maximal and minimal chamber areas. Consecutive 3 beats at each time point were analyzed. Ejection fraction obtained from both anterior-posterior and lateral projections was averaged and presented. All analyses were done in a blinded manner without knowledge of whether the test animal had been in the PBS or ICG-001 treated groups.

### Epicardium-Derived Cell (EPDC) Culture

Epicardial layers were removed from newborn mouse hearts, cut into small pieces, and then placed on gelatin coated plastic culture dishes with DMEM, 10% FBS and penicillin–streptomycin at 37°C in 5% CO2. After EPDC migrations from the original explants were observed at day 3, the media was changed to ICG-001, IQ1 or DMSO containing media.

### Quantification of *Wt1*-derived Cells in Embryonic Hearts

To label *Wt1*-derived cells in embryonic hearts with β-galactosidase, we crossed Wt1tm1 (EGFP/cre) Wtp/J with B6.129S4-Gt (ROSA)26Sortm1Sor/J mice (The JAX Laboratory). Pregnant females were treated with either DMSO or 0.5 M IQ1 solution in DMSO (0.25 ml/kg/day) orally from gestational day 12.5 for two days. Hearts from E14.5 embryos were dissected and fixed in 0.5% glutaraldehyde at 4°C over night. The hearts were stained for β-galactosidase activity at room temperature for 5 hours, re-fixed in 4% paraformaldehyde and embedded in paraffin blocks. Serial transverse sections were cut and counterstained with nuclear fast red solution (Electron Microscopy Science). One section of every 5 serial sections was photographed and a total of 10 images were obtained from each heart. Blue pixels (*Wt1*-derived cells) and red pixels (non-*Wt1* derived cells) were quantified by Photoshop7 software (Adobe). Control *n* = 13, IQ1 *n* = 8.

### Image Acquisition and Manipulation

Microscopic images were taken on an Axio Imager Z1 microscope, AxioCamHR camera and AxioVisionLE software or Leica DMI600B microscope, Leica DFC425 camera and Leica Application Suite software. Brightness and contrast were adjusted equally in comparable pictures with Photoshop7 software (Adobe).

### Statistical Analysis

Data are presented as mean ± standard error (s.e.m.). Differences in means between two experimental groups were analyzed using student’s t-test, assuming non-equal variances. *P*<0.05 was considered significant.

## Supporting Information

Figure S1 **Fibrotic area by histomorphometry.** Fibrotic area was quantified by Gomori’s trichrome staining. Blue green area was considered as fibrotic area. ICG-001 did not significantly change the fibrotic area at 7 and 10 days after post-coronary artery occlusion. Data are presented as mean ± SD.
(TIF)

Figure S2 **Left ventricular wall thickness by histomorphometry.** ICG-001 did not significantly change the LV thickness at 4 weeks post-coronary artery occlusion. Data are presented as mean ± s.e.m.
(TIF)

Figure S3 **qPCR analysis for cardiac hypertrophy markers.** ICG-001 did not change the significantly expression of *MCIP1, Nppa* and *Nhe1* post-coronary artery occlusion in rat heats. Data are presented as mean ± s.e.m.
(TIF)

Table S1 Primers used in quantitative PCR analysis.
(DOCX)

Text S1 **Supplemental experimental procedures.**
(DOCX)
