# Supplementary figures and images for: The Small Molecule Wnt Signaling Modulator ICG-001 Improves Contractile Function in Chronically Infarcted Rat Myocardium
Source: PLoS One. 2013 Sep 12;8(9):e75010. doi: 10.1371/journal.pone.0075010 (PMC3771968; doi:10.1371/journal.pone.0075010)

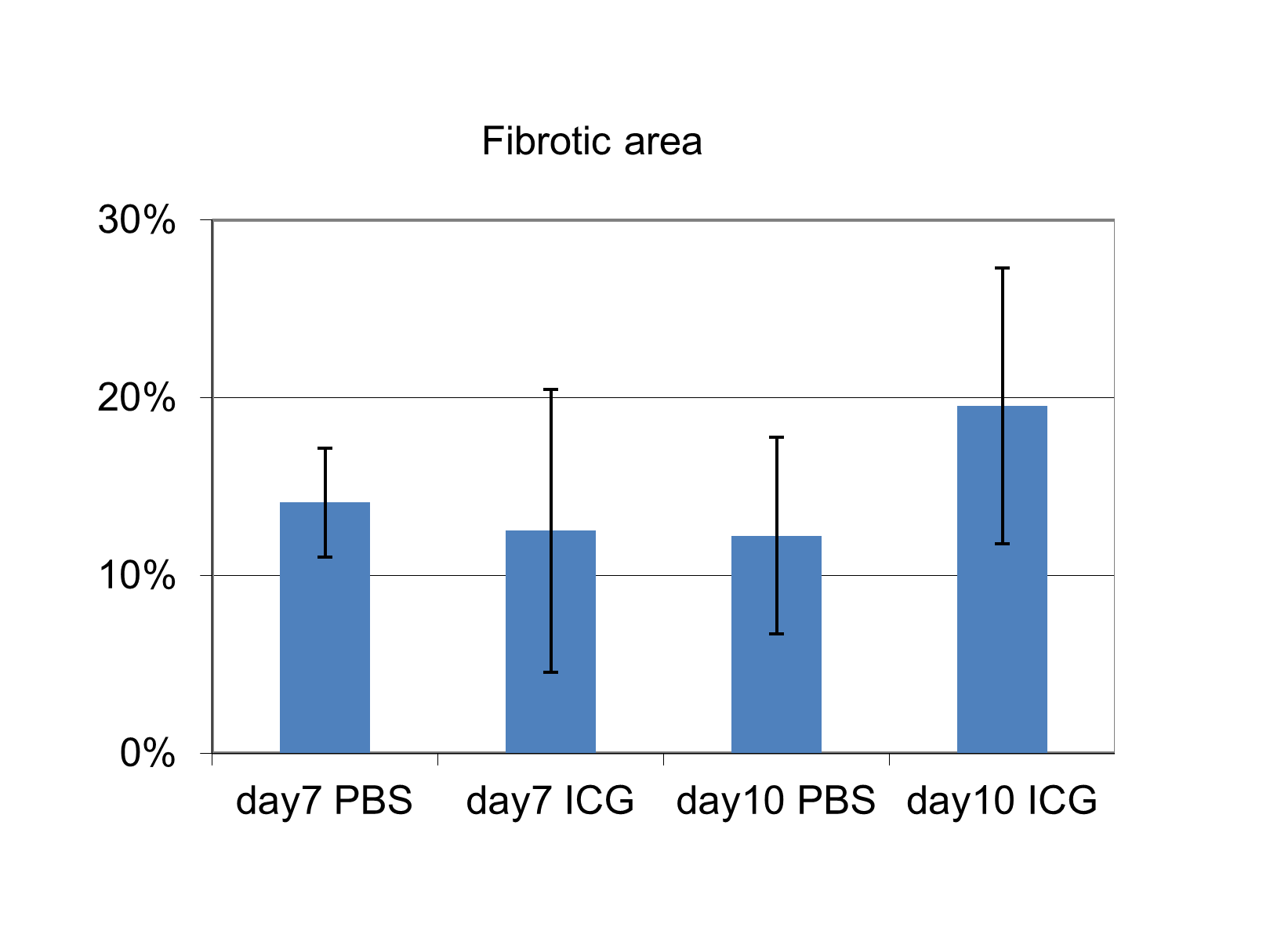

Supplement: Figure S1 — Fibrotic area by histomorphometry. Fibrotic area was quantified by Gomori’s trichrome staining. Blue green area was considered as fibrotic area. ICG-001 did not significantly change the fibrotic area at 7 and 10 days after post-coronary artery occlusion. Data are presented as mean ± SD. (TIF) [file pone.0075010.s001.tif]

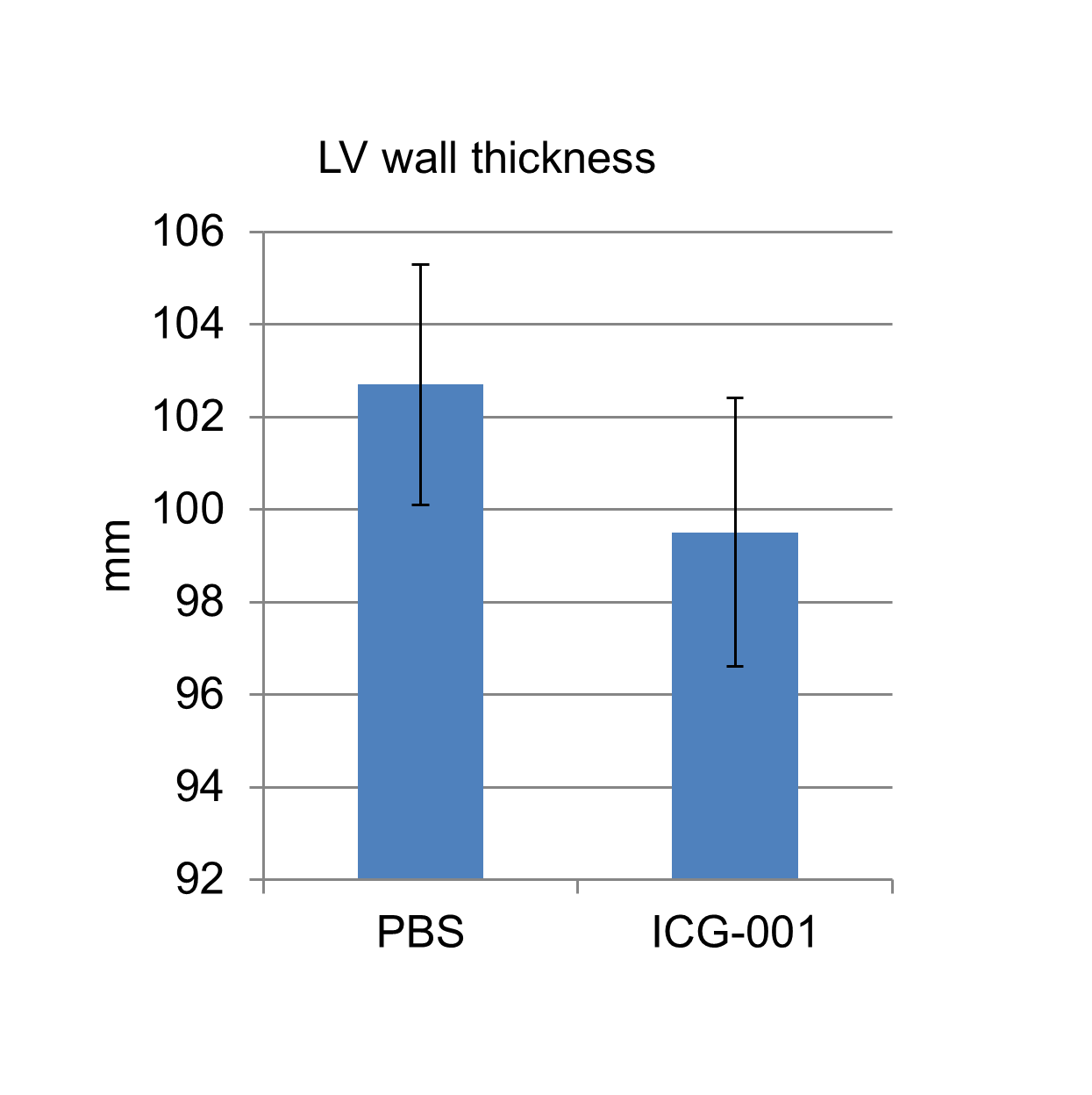

Supplement: Figure S2 — Left ventricular wall thickness by histomorphometry. ICG-001 did not significantly change the LV thickness at 4 weeks post-coronary artery occlusion. Data are presented as mean ± s.e.m. (TIF) [file pone.0075010.s002.tif]

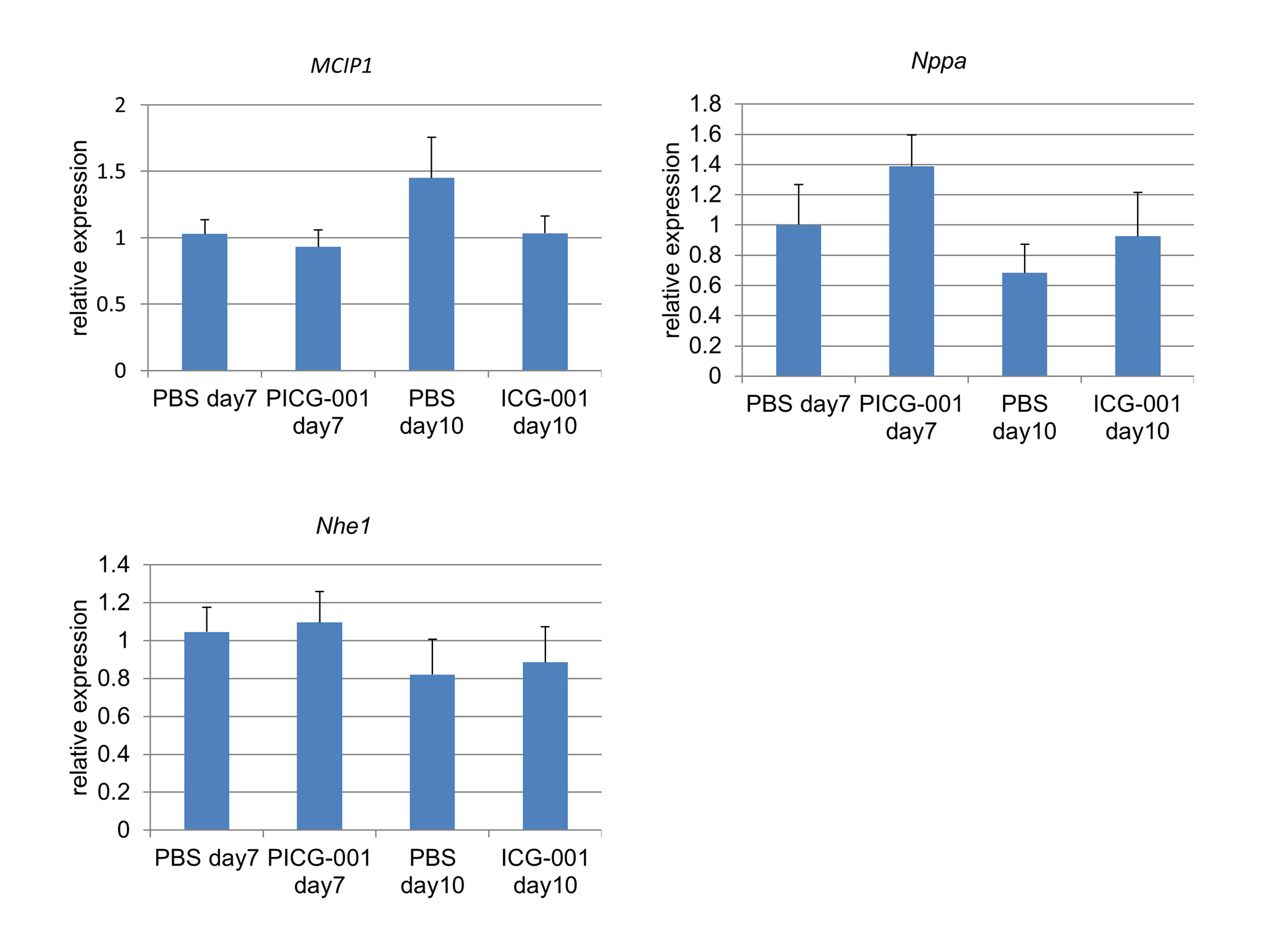

Supplement: Figure S3 — qPCR analysis for cardiac hypertrophy markers. ICG-001 did not change the significantly expression of MCIP1, Nppa and Nhe1 post-coronary artery occlusion in rat heats. Data are presented as mean ± s.e.m. (TIF) [file pone.0075010.s003.tif]
