# Supplementary material for: The Small Molecule Wnt Signaling Modulator ICG-001 Improves Contractile Function in Chronically Infarcted Rat Myocardium
Source: PLoS One. 2013 Sep 12;8(9):e75010. doi: 10.1371/journal.pone.0075010 (PMC3771968; doi:10.1371/journal.pone.0075010)
Supplement: Table S1 — Primers used in quantitative PCR analysis. (DOCX) [file pone.0075010.s004.docx]

**Table S1. Primers used in quantitative PCR analysis.**

| **species** | **Gene** | **Primer sequence (5’-3”)** |
| --- | --- | --- |
| rat | *Gapdh* | GTTACCAGGGCTGCCTTCTC, GGGTTTCCCGTTGATGACC |
| rat | *Gata4* | GCGGCCTCTACATGAAGCTC, CTTGGGCTTCCGTTTTCTG |
| rat | *Tnnt2* | CAGAGAGGAGGAAGGTGCTG, ACTCTGCCATAGCTCCTTGG |
| rat | *Kit* | CGTCGTACCAACCAAGACAG, AGAGCCAGCTCGTCATCTTC |
| rat | *Gdf15* | GCTTCCAGGACCTGCTGAG, GCACCTCTGGACTGAGTATCC |
| rat | *Fgf2* | TTTCCAAAACCTGACCCGATC, TGCAACTTTCTCCCTTCCTGC |
| rat | *Myh6* | GCTTTGGGAAGTTCATCAG, GCCTTTAGCTGGAAGATCAC |
| rat | *Myh7* | CTGAGGAGGCGGAGGAACAG, CTTGGCGCCAATGTCACG |
| rat | *Tmsb4x* | QuantiTect Primer Assay (QIAGEN) |
| rat | *Wt1* | GGAACCAGATGAACCTCGGAG, CGCTTCTCACTGGTTTCAGATGCTG |
| rat | *Nppa* | CCCGACCCACGCCAGCATGG, CAACTGCTTTCTGAAAGGGGT |
| rat | *Nppb* | ACAATCCACGATGCAGAAGCT, GGGCCTTGGTCCTTTGAGA |
| rat | *MCP1* | GTAACCCTTGAACCCCATT, CCATCCAATCGGTAGTAGCG |
| rat | *Nhe-1* | GCCCAATCCAGACAAACAGT, TGATTTTTGGCTTGGGTCTC |
| rat | *Ctgf* | TCGGGAGTCAGAACCTTGTC, GCTTGTTACCGGCAAATTCA |
| rat | *Vegfa* | TTGAGACCCTGGTGGACATC, CTCCTATGTGCTGGCTTTGG |
| rat | *Tbx5* | TCGCTGTGACTTCGTACCAG, TAACTCCAGCTGGTCACTGC |
| rat | *Vimentin* | GCACCCTGCAGTCATTGAGA, GCAAGGATTCCACTTTACGTT |
| mouse | *Gapdh* | GGTGCTGAGTATGTCGTGGA, ACAGTCTTCTGGGTGGCAGT |
| mouse | *cyclophilin A* | TTGGGCCGCGTCTCCTTCGA, GCCAGGACCTGTATGCTTCA |
| mouse | *Tbx18* | TGGGTTTGGAAGCTCTGGTG, CCTTGGAGCAGAGCTGAAGAAC |
| mouse | *Aldh1a2* | TTGCAGATGCTGACTTGGAC, TCTGAGGACCCTGCTCAGTT |
| mouse | *Tcf21* | GACCTTCAAGAGGTGGAGATGCT, CCTTCTGTGGAGACCCGTTCT |
| mouse | *Tmsb4x* | CACGAGCATTGCCTTCTTAT, TCTCTGCTAGCCAGACCATC |
| mouse | *Sdf1* | CGCTCTGCATCAGTGACGGTA, GTTCTTCAGCCGTGCAACAATC |
| mouse | *Wt1* | GCCTTCACCTTGCACTTCTC, GACCGTGCTGTATCCTTGGT |
| mouse | *Gdf15* | AGACTGTGCAGGCAACTCTTGA, ACACTCGCCCACGCACAT |
| mouse | *Kit* | TCATCGAGTGTGATGGGAAA, GGTGACTTGTTTCAGGCACA |
