# Supplementary material for: The Small Molecule Wnt Signaling Modulator ICG-001 Improves Contractile Function in Chronically Infarcted Rat Myocardium
Source: PLoS One. 2013 Sep 12;8(9):e75010. doi: 10.1371/journal.pone.0075010 (PMC3771968; doi:10.1371/journal.pone.0075010)
Supplement: Text S1 — Supplemental experimental procedures. (DOCX) [file pone.0075010.s005.docx]

**Text S1. Supplemental experimental procedures.**

**Rat EMC Culture**

The EMC rat epicardial cell line (epicardial mesothelial cells) was kindly provided by Dr. Henry Sucov, University of Southern California. The EMC cells were maintained in DMEM supplemented with 10% FBS (fetal bovine serum) and penicillin–streptomycin at 37 °C in 5% CO2.

**Quantitative PCR**

RNA was extracted with TRIzol Reagent (Invitrogen), quantified by NanoDrop (Thermo Scientific) and reverse-transcribed to cDNA using an iScript cDNA Synthesis Kit (Bio-Rad). Quantitative PCR (qPCR) was carried out using a MyiQ system and iQTM SYBR® Green Supermix (Bio-Rad). Primers are listed in Table S1. Reaction volumes of 25 µl were used with 2 µl of cDNA and 0.6 μM of primers. The results were normalized to the expression level of the housekeeping genes, *Gapdh* or *cyclophilin A*, which were quantified simultaneously with the target gene. Data were analyzed by the iQ5 optical system software (Bio-Rad) and the 2^-∆∆CT method was utilized.

**Co-immunoprecipitation (co-IP) Assay**

Rat EMC cells were treated with DMSO control, 5 μM IQ1 or 10 μM ICG-001 for 24 hours. After treatment, cells were harvested and nuclear lysates were prepared for co-immunoprecipitation using anti-CBP (Santa Cruz, cat. #: sc-369) or anti-p300 (Santa Cruz, cat. #: sc-584) and immunoblotted for β-catenin (BD, cat. #:610153).

**TOPFlash Assay**

Rat EMC cells were co-transfected with super FOPFlash or super TOPFlash and renilla luciferase pRL-TK vectors. The cells were subsequently treated with DMSO control, 10 μM IQ1 or 10 μM ICG-001 for 24 h. After treatment, luciferase activities were measured and normalized to renilla luciferase activity.

**Heart Explant Organ Culture**

Hearts were dissected from E12.5 or E13.5 mouse embryos and placed front side up on Polycarbonate Membrane Filters (Whatman) floating in DMEM media (Cellgro) with 10% FBS and penicillin–streptomycin at 37 °C in 5% CO2. DMSO, ICG-001 or IQ1 were added to the culture media to treat the heart explants. The explants in each treatment continued to beat throughout the culture period. After 5 or 24 hours, the ventricles were removed and subjected to RNA extraction.

**Immunocytochemistry (ICC)**

Cultured cells were fixed in 4% paraformaldehyde for 10 min at 4 °C, washed in PBS, permeabilized in 0.1% Triton X-100, and blocked in 1% BSA(bovine serum albumin) in PBS for 30 min before incubation with antibodies. Antibodies and solution used were FITC Mouse Anti-β-catenin antibody (1:400, BD Transduction Laboratories), Anti-WT1 antibody ( 1:100, Santa Cruz, sc-192), rhodamine-phalloidin (1:200, Invitrogen), Anti-Actin or α-Smooth Muscle antibody (1:2000, Sigma, A2558), Alexa Fluor 488 anti-mouse IgG (1:200, Invitrogen), Alexa Fluor 568 anti-rabbit IgG (1:200, Invitrogen), and VectaShield solution containing DAPI (Vector). The images were captured using an AxioImager Z1 and AxioVision digital image processing software (Carl Zeiss).

**IQ1 Administration to Mouse Embryos**

Timed-pregnant female ICR mice were given IQ1 (0.5 M) solution in DMSO or DMSO as control in the diet 0.25 ml/kg/day for 1-4 days before embryo isolation. Dissected embryonic hearts were fixed in 4% paraformaldehyde and embedded in paraffin for H-E transverse sections.

**Whole Mount Immunohistochemistry**

Fixed embryonic hearts were dehydrated and rehydrated with methanol, blocked in 1% BSA, incubated with anti-PECAM-1 antibody (1:200 Santa Cruz, sc-1506) over night at 4 °C, washed in PBS, incubated with HRP-conjugated anti-goat IgG (Santa Cruz) and colorized with diaminobenzidine (DAB) substrate (Sigma).

**Left Ventricular Cavity Volume and Histomorphometric Assessments**

After angiographic analysis, hearts were arrested with a bolus injection of potassium chloride. The hearts were removed and pressure-fixed with 10% phosphate-buffered formalin for 2 hours and stored for analysis of left ventricular cavity volume. Size of the left ventricular cavity was determined by filling the ventricular cavity with distilled water. The hearts were weighed with and without water, and the differences were calculated as the left ventricular cavity volume. Means of the 3 measurements are presented. For histomorphometric analysis, the formalin-fixed specimens were sliced into 3 pieces and embedded in paraffin. Serial sections (5 μm) were prepared from the specimens and stained with picrosirius red for collagen depositions. The histological images were photographed and analyzed using the ImageJ software (NIH, version 1.38). Scar thickness and left ventricular circumference (epi- and endocardial circumferences/2) were measured. Infarct size, ventricular wall thinning ratio (left ventricular infarct wall thickness/remote wall thickness), and expansion index ((left ventricular cavity/total left ventricular area) × (infarct wall thickness/remote wall thickness)) were calculated.

**Cardiomyocyte Regeneration Analysis**

Rats were given bromodeoxyuridine (BrdU, 1 mg/ml) in their drinking water from the day of surgery for 7 days. The rats were euthanized at 7 and 10 days post-surgery. The hearts were removed. A cross-sectional cut was made to equally divide the infarcted area. A section toward the apex was rapidly stored in liquid N_2_ for RNA analysis, and the other half for staining for BrdU incorporation. BrdU incorporation in the infarct area was determined using a BrdU staining kit (Invitrogen). Gomori’s trichrome staining was performed using the Chromaview stain kit, Gomori’s trichrome, green collagen (Thermo Fisher Scientific).
